# Red blood cell transfusion in the critically ill patient

**DOI:** 10.1186/2110-5820-1-43

**Published:** 2011-10-04

**Authors:** Christophe Lelubre, Jean-Louis Vincent

**Affiliations:** 1 Department of Intensive Care, Erasme Hospital, Université libre de Bruxelles, Route de Lennik 808, 1070 Brussels, Belgium

## Abstract

Red blood cell (RBC) transfusion is a common intervention in intensive care unit (ICU) patients. Anemia is frequent in this population and is associated with poor outcomes, especially in patients with ischemic heart disease. Although blood transfusions are generally given to improve tissue oxygenation, they do not systematically increase oxygen consumption and effects on oxygen delivery are not always very impressive. Blood transfusion may be lifesaving in some circumstances, but many studies have reported increased morbidity and mortality in transfused patients. This review focuses on some important aspects of RBC transfusion in the ICU, including physiologic considerations, a brief description of serious infectious and noninfectious hazards of transfusion, and the effects of RBC storage lesions. Emphasis is placed on the importance of personalizing blood transfusion according to physiological endpoints rather than arbitrary thresholds.

## Introduction

Red blood cell (RBC) transfusion is commonly required in critically ill patients. Several recent, observational, multicenter studies reported that approximately one third of critically ill patients received a blood transfusion at one time or another during their stay in the intensive care unit (ICU) (Table 1). Because of the frequent use of this intervention, it is important for the ICU physician to be aware of recent developments in this continuously evolving field of medicine. In this narrative review, we consider some key aspects of transfusion medicine in the ICU, focusing on aspects relevant to the critically ill patient, including prevalence and reasons for blood transfusion, epidemiology and etiology of anemia in these patients, pathophysiological considerations on tolerance to anemia, and efficacy of RBC transfusion. Safety concerns, including questions of RBC storage and leukoreduction, are then discussed, followed by a proposal for an integrated approach to transfusion decisions and a discussion on economic aspects and alternatives to blood transfusion.

**Table 1:** Multicenter observational studies of transfusion in general ICU patients

Author	Year study was conducted	Country/region	No. of patients and number of ICUs	Percentage transfused in ICU	Pretransfusion hemoglobin level	Mean no. of units transfused in ICU	Mean age of blood (days)
Hebert et al. [9]	1993	Canada	5,298 patients in 6 ICUs	25.0	Mean: 8.6 ± 1.3 g/dl	NS	NS
Vincent et al. [3]	1999	Western Europe	3,534 patients in 146 ICUs	37.0	Mean: 8.4 ± 1.3 g/dl	4.8 ± 5.2	16.2 ± 6.7
Rao et al. [6]	1999	UK	1,247 patients in 9 ICUs	53.0	Median: 8.5 (IQR: 7.9-9) g/dl	6.75 (hemorrhage) and 4.25 (anemia)	NS
Corwin et al. [5]	2000 - 2001	USA	4,892 patients in 284 ICUs	44.0	Mean: 8.6 ± 1.7 g/dl	4.6 ± 4.9	21 ± 11.4
Walsh et al. [7]	2001	UK (Scotland)	1,023 patients in 10 ICUs	39.5	Median: 7.8 (7.3-8.5) g/dl	Mean: 1.87 unit/ICU admission	NS
French et al. [10]	2001	Australia and New Zealand	1,808 patients in 18 ICUs	19.8	Median: 8.2 (range: 4.4-18.7) g/dl	Mean: 4.18	NS
Vincent et al. [34]	2002	Western and Eastern Europe	3,147 patients in 198 ICUs	33.0	Median: 8.2 g/dl	5.0 ± 5.8	NS
Westbrook et al. [8]	2008	Australia and New Zealand	5,128 patients in 47 ICUs	14.7	Mean: 7.7 g/dl	Median: 2 (IQR: 1-4)	Median: 14 (IQR: 9.5-21.5)

*ICU *intensive care unit; *NS *not specified; *IQR *interquartile range

## Epidemiology of anemia and red blood cell transfusion in the ICU

Anemia is common in ICU patients and appears early in the ICU course [1]. In an observational, multicenter, cohort study in Scotland, 25% of patients admitted to the ICU had a hemoglobin level < 9 g/dl [2]. Similar results were reported in the ABC study [3], in which 29% of patients had a hemoglobin concentration < 10 g/dl on admission. Even in nonbleeding ICU patients, hemoglobin levels tend to decrease early [3]. This decrease is more pronounced in septic than in nonseptic patients [4], at least in part because of their inflammatory response; more frequent blood sampling may also contribute.

Interestingly, anemia and the need to restore adequate oxygen delivery (DO_2_) are the most common indications for transfusion, rather than acute bleeding [3,5-10]. Anemia in the critically ill patient is a multifactorial phenomenon that has been compared to the so-called "anemia of chronic illness" [11]. Apart from evident causes of anemia, such as primary blood losses (e.g., trauma, surgery, gastrointestinal bleeding), multiple other etiologies contribute to its pathophysiology and often coexist in the same patient [11]. These include blood losses related to minor procedures or phlebotomy, and hemodilution secondary to fluid resuscitation. Some studies have suggested that blood sampling may average as much as 40 ml/day [3,4], but the amount of blood required may decrease with technological developments in analytic methods. Other mechanisms for anemia include an inflammatory response with blunted erythropoietin (EPO) production, abnormalities in iron metabolism, and altered proliferation and differentiation of medullar erythroid precursors [11]. As a consequence, RBC deformability is decreased [12], whereas RBC adherence to the endothelium is increased, especially in septic patients, potentially leading to microcirculatory impairment and tissue hypoxia [13].

## Tolerance to anemia in healthy subjects and in the critically ill patient

Tolerance to anemia is highly dependent on the volume status of the patient, physiological reserve, and the dynamics of the anemia (for example, chronic, such as the anemia of sepsis, versus acute, such as hemorrhagic conditions). Normovolemic anemia is better tolerated than anemia in hypovolemic states (e.g., acute bleeding in trauma patients or surgery) in which cardiac output acutely decreases. In healthy subjects submitted to normovolemic hemodilution, cardiac output increases because of decreased blood viscosity (especially relevant in severe anemia) and increased adrenergic response, allowing tachycardia and increased myocardial contractility. Other phenomena include blood flow redistribution (to heart and brain) and an increased oxygen extraction ratio (reflected by a decrease in mixed venous saturation [SvO_2_]). These mechanisms allow healthy humans to tolerate severe degrees of normovolemic anemia [14,15], although side effects, such as arrhythmias or ST changes, can be observed in extreme cases [16,17]. The myocardium is the organ at risk in cases of acute anemia in which both tachycardia and increased ventricle contractility may increase myocardial oxygen demand. Because myocardial oxygen extraction is already almost maximal at rest, every increase in myocardial oxygen demand must be accompanied by increased coronary blood flow [18]. This can become problematic in patients with stenotic coronary arteries especially when tachycardia is present, which can decrease diastole-dependent left ventricle perfusion.

Therefore, in critically ill patients, especially those with heart failure or coronary artery disease (CAD), the myocardium may not tolerate such low hemoglobin levels [19]. In acute myocardial infarction, anemia may worsen myocardial ischemia, generate arrhythmias, and potentially increase infarct size [20]. In patients with acute coronary syndrome or heart failure, anemia increases morbidity and mortality [21,22]. For these reasons, patients with cardiac problems should be managed with a more liberal approach to transfusion than other patients [23,24].

## Purpose and efficacy of blood transfusion

The primary purpose of blood transfusion is to increase DO_2_, which is determined by cardiac output and arterial content of oxygen, the latter being dependent on the hemoglobin level. Hence, blood transfusions can, theoretically at least, limit tissue hypoxia [13,25,26]. But does this really happen in clinical practice? It is obvious that RBC transfusions can be lifesaving in situations of acute severe anemia or in bleeding patients in whom RBC administration can increase both oxygen arterial content and cardiac output. However, in the absence of bleeding, the increase in hemoglobin concentration could very well be offset by a decrease in cardiac output because of the increase in blood viscosity associated with a decreased sympathetic response [27,28]. DO_2 _has been shown to increase following RBC transfusion in numerous studies [26], but not in all [29].

The effects of RBC transfusion on the relationship between DO_2 _and oxygen uptake (VO_2_) are even more difficult to predict. Some studies reported that VO_2 _increased following RBC transfusion, whereas others did not [26], and variable effects have been reported on tissue perfusion as assessed by gastric mucosal pH or near-infrared spectroscopy (NIRS) [30]. The reasons for these contradictory results lie primarily in the degree of severity of hypoxia preceding the RBC transfusion [31], which influences the dependency of VO_2 _on DO_2_. Methodological problems (imprecision in determination of VO_2_, assessment of global VO_2 _instead of regional VO_2_, poor correlation between systemic oxygenation parameters, and oxygenation in the microcirculation [13]) also may contribute to these discrepancies [31].

## Safety concerns of blood transfusions

### Impact on outcome

Red blood cell transfusions have been associated with worse outcomes in several populations of patients, including critically ill patients. In a recent systematic review of 45 observational studies reporting the impact of transfusions on patient outcome (mortality, infections, acute respiratory distress syndrome [ARDS]) in populations of trauma, general surgery, orthopedic surgery, acute coronary syndrome, and ICU patients, Marik and Corwin [32] identified RBC transfusion as an independent predictor of death (pooled odds ratio (OR) from 12 studies, 1.7; 95% confidence interval (CI), 1.4-1.9), infectious complications (pooled OR from 9 studies, 1.8; 95% CI, 1.5-2.2), and ARDS (pooled OR from 6 studies, 2.5; 95% CI, 1.6-3.3). In ICU patients, the three studies included in the review (ABC study [3], CRIT study [5], and a study by Gong et al. [33]) consistently showed a statistically significant association of RBC transfusion with mortality.

On the other hand, analysis of data from a multicenter, prospective, observational study of 3,147 patients in 198 European ICUs (the SOAP study) indicated that blood transfusions were not associated with increased mortality by multivariate analysis or propensity matching [34]. In contrast, an extended Cox proportional hazard analysis showed that patients who received a transfusion in fact had a better survival, all factors being otherwise equal. An increased rate of transfused leukoreduced RBCs reported in this study (in which 76% of centers routinely used leukoreduced RBCs) could perhaps account for the differences between the earlier ABC study [3] (in which 46% of centers used leukodepleted blood most of the time) and the SOAP study [34]. It also is possible that transfusion thresholds have become so low that the benefits of blood transfusion outweigh the risks.

In patients with acute coronary syndrome, several studies have shown poorer outcomes, including increased mortality, in transfused groups compared with nontransfused patients after adjustment for potential confounders [21,35-37]; similar findings have been reported in patients who undergo percutaneous coronary interventions (PCI) [38]. However, although still controversial, RBC transfusions may be useful in subgroups of elderly patients with acute myocardial infarction [39] or patients with ST elevation myocardial infarction (STEMI) [21].

Patients who undergo cardiac surgery seem to have worse outcomes when transfused, including higher mortality [40,41], increased occurrence of postoperative infections [41,42], increased time on mechanical ventilation [40,43], and higher incidence of postoperative acute kidney injury [41,44].

Other studies have reported that trauma patients [45,46], including those with burns [47], may have increased mortality rates associated with receiving blood transfusions. In contrast, RBC transfusion has been reported to be associated with improved outcomes in patients with traumatic brain injury or subarachnoid hemorrhage [48,49]. In the early resuscitation of patients with severe sepsis, implementation of a therapeutic protocol that included RBC transfusion to obtain a hematocrit > 30% was associated with a significant reduction in hospital mortality [50].

These results should be interpreted with caution, because most of these data come from observational, retrospective studies, which are subject to numerous biases and sometimes control poorly for confounders, despite the use of various statistical tools, such as logistic regression [51]. It is clear that analyses should not include only admission data. For example, in a well-defined patient population, such as after cardiac surgery, patients who develop gastrointestinal bleeding and require a blood transfusion have a worse prognosis, which is not necessarily the result of the blood transfusion. It is of paramount importance that all risks factors are taken into account. Ruttinger et al. [52] illustrated this point very well. In a series of more than 3,000 surgical patients, these authors showed by using a limited multivariable analysis that transfusions were associated with a worse outcome, but a more complete analysis cancelled out this statistical observation.

### Noninfectious serious hazards of transfusions

The reasons for the apparent worse outcome of transfused compared with nontransfused critically ill patients may be found in several detrimental effects of transfused blood, globally referred to under the acronym "Non-Infectious Serious Hazards Of Transfusion" or NISHOT (Table 2) [53]. These include, among others, deleterious effects on the immune system (transfusion-related immunomodulation or "TRIM") or on the cardiopulmonary system, e.g., transfusion-related acute lung injury ("TRALI") [54] or transfusion-associated circulatory overload ("TACO"); the latter is currently the leading reported cause of transfusion-associated mortality [55]. These effects may be enhanced by pathologic conditions (e.g., sepsis) in which the microcirculation is impaired [56] and/or when the RBCs have been stored for some time.

**Table 2:** Selected infectious and non-infectious hazards of RBC transfusion in the ICU environment

		Estimated frequency (event/no. of transfusions)*	Comment
Infectious transmission [89,90]			
	HIV	1/2.3 10^6^	
	HBV	1/350000	
	HCV	1/1.8 10^6^	
	HTLV 1/2	1/2 10^6^	
	Bacterial contamination	1/14,000 to 1/28,000	GNB such as *Y. Enterocolitica *mostly encountered
Noninfectious complications			
Immune-mediated [53,89]			
	Acute hemolytic transfusion reactions	1/10,000 to 1/50,000	Most frequently due to IgM, sometimes IgG
	Febrile nonhemolytic transfusion reactions	1/500	Reduced incidence with prestorage leukoreduction
	Anaphylactic reactions	1/20,000 to 1/50,000	May be associated with IgA deficiency
	Transfusion-related acute lung injury (TRALI)	Highly variable (e.g., 1/29,000 [91], 1/46,700 [92], 1/173,000 [93] units transfused)	Must be differentiated from TACO
	Posttransfusion purpura	1/143,000	Rare; occurs 5-10 days after transfusion
	Transfusion-associated graft versus host disease	Rare (prevention by irradiation of blood products)	Mostly in immunocompromised hosts, poor prognosis
Nonimmune-mediated [89,94]			
	Incorrect blood component transfused (IBCT)	9.7/100,000 components	Remains frequent despite prevention strategies; must be differentiated from near-miss transfusion
	Transfusion-associated circulatory overload (TACO)	Up to 1% of transfusions	Major cause of transfusion-related death
	Hyperkalemia		Mainly after transfusion in newborns
	Hypocalcemia - hypothermia		Mainly after massive transfusion
	Dilutional coagulopathy/thrombocytopenia		Mainly after massive transfusion

*HIV *human immunodeficiency virus; *HBV *hepatitis B virus; *HCV *hepatitis C virus; *HTLV *human T lymphotropic virus; *GNB *Gram-negative bacteria

*Frequencies may vary among studies and are only indicative

### Question of RBC storage

During storage, RBCs undergo a series of biological and biochemical changes collectively referred to as "the storage lesion" [57]. This includes intracellular changes (progressive depletion of 2,3-diphosphoglycerate [2,3-DPG] with increased affinity of hemoglobin for oxygen, depletion of ATP), membrane changes (membrane vesiculation, morphological changes eventually leading to irreversibly deformed spheroechinocytes, lipid peroxidation and increased expression of phosphatidylserine, decreased deformability), and changes in the storage medium (decreased pH, increased potassium, release of proinflammatory cytokines). These stored RBCs also have an increased tendency to adhere to endothelium and could promote vasoconstriction; the stored RBCs act as a "sink" for nitric oxide [58]. Some animal studies [13] have shown deleterious effects of old RBCs on the microcirculation (potentially leading to tissue hypoxia and organ dysfunction). A human study found an inverse correlation between the age of transfused RBCs and maximal change in gastric mucosal pH, but these findings were challenged in subsequent studies [59-61].

The clinical consequences of storage lesions are still not clear. A recent review of the literature [57] identified 24 studies that address the effects of RBC length of storage on clinical (mortality, infections, length of stay, length of mechanical ventilation) or physiological (microcirculation, gastric mucosal pH) endpoints. Some studies found associations between the age of transfused RBCs and poorer outcomes, whereas others did not. Overall, no clear detrimental effect of RBC age could be identified; however, definitive conclusions are difficult to obtain because of numerous statistical limitations and biases inherent to the study designs [51,62]. Several, large, randomized, controlled trials in adult ICU and cardiac surgery patients are currently ongoing to address the clinical relevance of RBC storage. In the multicenter, double-blind prospective ABLE (Age of Blood Evaluation) study [63], adult patients admitted to the ICU are randomly assigned to receive leukoreduced RBCs stored for less than 7 days or issued according to standard procedure (expected average storage time of 19 days). The primary endpoint of this study is 90-day all-cause mortality. The target number of patients is 2,510 (for an expected improvement in primary endpoint greater than 5%) with an anticipated completion date by April 2013. The Red Cell Storage Duration Study (RECESS) is a multicenter, randomized study in patients (age 12 years and older) who undergo complex cardiac surgery and are likely to require RBC transfusion [64]. Patients who need transfusion are randomized to receive RBCs stored for ≤ 10 days or ≥ 21 days. The primary endpoint of this study is the change in the Multiple Organ Dysfunction Score (MODS) from baseline to day 7, with secondary outcomes including all-cause 28-day mortality. The target number of patients is 1,832, and the anticipated completion date is September 2013.

The results of these trials, especially if older blood appears to be harmful, could have important logistic implications for blood banks [65,66].

### Question of leukoreduction

Many of the adverse effects associated with the transfusion of allogeneic RBCs have been shown to be related to the infusion of white blood cells (WBCs) present in the blood product. Leukoreduction is a process in which WBCs are reduced in number through centrifugation or filtration [67]. This process allows removal of approximately 99.995% of WBCs, but several thousand leukocytes (0.005% of a 500 ml blood unit) may still be present in the processed blood [67]; hence, the word "leukoreduction" is better than "deleukocytation." The beneficial effects of this process include decreased febrile nonhemolytic transfusion reactions, decreased transmission of certain pathogens, such as Epstein-Barr virus (EBV) or cytomegalovirus (CMV), parasites and prions [67], and possibly decreased lung injury, such as TRALI. Moreover, prestorage leukoreduction, in which WBC removal occurs before RBC storage, avoids the need for a leukodepletion filter during transfusion [67] (but a 170-200-μm filter still needs to be incorporated into the intravenous blood line).

In several studies, prestorage leukoreduction decreased RBC storage lesions, with fewer immunomodulating properties [68] and less adhesion of stored RBCs to the endothelium [69]. A clinical benefit of leukoreduction is still somewhat controversial, particularly in the critically ill patient where no randomized, controlled trial has been performed [70]. In a before-after study of 14,786 patients who underwent cardiac surgery, repair of hip fracture, or who required intensive care after surgery, there was a 1% decrease in mortality rate associated with the implementation of universal leukoreduction [71]. In a recent meta-analysis of nine RCTs involving 3,093 surgical patients, the use of leukoreduction significantly reduced the odds of postoperative infection (summary OR, 0.522; 95% CI, 0.332-0.821; *p *= 0.005) [72]. This observation had been suggested in a previous meta-analysis [73] but has been challenged by another recent meta-analysis [74]. Nevertheless, leukoreduction makes sense, and many countries have adopted it as routine, even though costs are elevated. In Europe, at the time of the SOAP study in 2002, 76% of centers reported using leukodepleted blood routinely [34], whereas an earlier study performed in the same countries reported lower rates [3].

## The decision to transfuse

Classically, the decision to transfuse is driven by arbitrary "triggers" (hemoglobin level) rather than clinical or physiologic findings. Data from the CRIT study [5], in which there was little evidence that age or comorbidities significantly influenced transfusion practice, tend to support this view.

Current recommendations for RBC transfusion [75,76] are mainly based on the famous "TRICC" (Transfusion Requirements In Critical Care) trial in which patients assigned to a restrictive transfusion strategy (transfusion if hemoglobin level < 7 g/dl) had similar 30-day mortality rates (and even lower mortality in subgroups with APACHE II < 20 and patients younger than age 55 years) than patients transfused according to a more liberal strategy (transfusion if hemoglobin level < 10 g/dl) [77]. In cardiac surgery patients, the recent randomized, monocenter "TRACS" (Transfusion Requirements after Cardiac Surgery) trial, which compared a restrictive to a liberal strategy (transfusion when hematocrit < 24% or < 30%, respectively), reported no difference in the primary endpoint (composite of 30-day mortality and morbidity [cardiogenic shock, ARDS, acute kidney injury]) between the groups [78].

However, it is quite clear there is no "magic" hemoglobin or hematocrit trigger, and for the same level of hemoglobin, some patients will do well, whereas others will not. Thus, the decision to transfuse a patient should be individualized, taking into account several factors, including signs and symptoms of tissue hypoxia (angina pectoris, cognitive dysfunction diagnosed by neuropsychological tests, or increased P300 latencies [79-81]), increased blood lactate levels [82], or electrocardiographic changes suggestive of myocardial ischemia.

Indirect measures of oxygenation, such as a decreased SvO_2 _or central venous oxygen saturation (ScvO_2_), also may be considered [82]. For example, in a study of early goal-directed therapy in patients with severe sepsis or septic shock admitted to an emergency department, a decrease in ScvO_2 _< 70% initiated a therapeutic intervention, including fluid resuscitation, inotropes, vasopressors, and RBC transfusion to increase hematocrit to > 30% [50]. Use of a decreased ratio of cardiac index to oxygen extraction (CI/EO_2 _ratio) may be better, because this parameter also reflects the cardiac response to anemia [83].

## Economic aspects of blood transfusion

The costs of blood transfusion are particularly complex to assess because of the many factors that have to be taken into consideration (blood collection and screening for pathogens; blood component processing, including leukoreduction, storage, transport to the transfusion facility; administration of blood to the patient; management of potential short- and long-term transfusion-related side effects) [84]. The subtype of the blood unit also may play a role because some products, such as CMV-negative or autologous units, are costlier than classical allogeneic RBCs. Consequently, studies in this field have given extremely varied results, which are not easily comparable.

Evidence has shown increased costs of RBC transfusion over time [85], related to various factors, including (but not limited to) use of leukoreduction and more sophisticated methods for pathogen detection, such as nucleic acid testing (NAT) [84]. For example, a study in Canada evaluated the mean societal cost of one allogeneic RBC unit at 264.81 US$, twice the cost estimated 7 years earlier [86]. Generally, these reported values are probably underestimated, and some have calculated that the cost of blood to society could in fact be twofold higher [84].

## Alternatives to blood transfusion

Because of limited availability, costs and safety concerns related to blood transfusion, several strategies to reduce blood transfusions can be considered in addition to increasing transfusion trigger thresholds. These include approaches to reduce blood losses, for example use of antifibrinolytic agents, such as tranexamic acid or epsilon-aminocaproic acid (EACA) and techniques of cell salvage during surgery; also, the use of small volume sample tubes can limit the blood losses related to sampling for laboratory studies. In a meta-analysis of 9 randomized controlled trials [87], subcutaneous administration of recombinant erythropoietin (EPO) in critically ill patients was shown to be associated with decreased transfusion rates, but this was not associated with improved mortality (except possibly in a subgroup of trauma patients [88]). Concerns also have been raised about potentially increased rates of deep vein thrombosis [88]. The development of artificial oxygen carriers is under investigation, but these have their own problems [89]. Further research is needed to improve these alternative strategies.

## Conclusions

RBC transfusion can be lifesaving. During the past two decades, however, safety concerns have emerged, with suggestions that morbidity and mortality may be increased in patients who receive blood transfusions. Therefore, the decision to transfuse should be individualized, based on a rational approach and taking into account physiologic variables in addition to the hemoglobin value. This strategy, along with the use of alternatives whenever possible to limit bleeding, should limit unnecessary exposure to RBCs.

## Competing interests

The authors declare that they have no competing interests.

## Authors' contributions

CL drafted the manuscript. The manuscript was revised for intellectual content by JLV. Both authors read and approved the final manuscript.
